# Meet the Country Editor

**DOI:** 10.2174/1570159X2109230518114245

**Published:** 2023-07-10

**Authors:** Albert Fernández-Teruel

**Affiliations:** 1 Department of Psychiatry & Forensic Medicine, Autonomous University of Barcelona, Barcelona, Spain

Prof. Alberto Fernández-Teruel is Associate Professor at the Department of Psychiatry & Forensic Medicine (Medical Psychology Unit) of the Universidad Autónoma de Barcelona (UAB) and Director of the Animal Research Laboratory of this Unit (“Animal and Human models of mental disorders” research group) since 2004. He received his PhD from UAB in 1989, and developed pre- and postdoctoral work at the University of Cagliari (Italy) and the ETH-Zentrum (Zürich, Switzerland) and the University of Cagliari, where he has also been Visiting Professor/Scientist. He has published over 150 peer-reviewed articles. Part of his research focuses on neurogenetics, quantitative genetics and neurobiology of anxiety, schizophrenia-relevant models, and the impact of early experience in these animal models.
